# Structure Identification of Uncertain Complex Networks Based on Anticipatory Projective Synchronization

**DOI:** 10.1371/journal.pone.0139804

**Published:** 2015-10-07

**Authors:** Liu Heng, Wang Xingyuan, Tan Guozhen

**Affiliations:** Faculty of Electronic Information and Electrical Engineering, Dalian University of Technology, Dalian, China; Tianjin University, CHINA

## Abstract

This paper investigates a method to identify uncertain system parameters and unknown topological structure in general complex networks with or without time delay. A complex network, which has uncertain topology and unknown parameters, is designed as a drive network, and a known response complex network with an input controller is designed to identify the drive network. Under the proposed input controller, the drive network and the response network can achieve anticipatory projective synchronization when the system is steady. Lyapunov theorem and Barbǎlat’s lemma guarantee the stability of synchronization manifold between two networks. When the synchronization is achieved, the system parameters and topology in response network can be changed to equal with the parameters and topology in drive network. A numerical example is given to show the effectiveness of the proposed method.

## Introduction

During the past decades, complex networks have attracted lots of attention in scientific and technological fields including mathematics, physics, engineering, biological sciences, and so on [1–4]. There are a lot of issues about complex networks which have been investigated by now such as the characteristics about small-world and scale-free [5, 6], the analysis of dynamics and topologies about networks [7–12], various synchronization in complex networks [13–24], and so on. In these fields, there always exist some networks which have unknown parameters or uncertain topological structure need to be identified. For example, it is very important to find the faulty spot or the failing edge in time when a mistake occurs in a power network, a communication network, or in Internet. Thus, the research of identification about complex network is of theoretical and practical importance.

So far, a few methods have been proposed for topological identification of complex networks [25–27]. Nowadays, using the dynamical character of complex networks, such as synchronization, to identify unknown parameters or uncertain topology structure, has been studied more and more widely. Zhao and others investigated an adaptive feedback laws to identify the extract topology of weighted complex dynamical networks with and without time delays [28]. In their paper, they used PE conditions to guarantee the effeteness of their method. Liu and others investigated a novel adaptive feedback control method to simultaneously identify the unknown or uncertain time delay complex networks structure or system parameters [29]. Chen and others described how a network can practically be identified by an adaptive-feedback control algorithm [30]. They found that the linear independence condition of the coupling terms proposed in this brief is necessary and sufficient for network identification, and synchronization is a property of a dynamical network that makes identification of the topology of the network impossible. Che and others studied two kinds of synchronization based topology identification of uncertain complex networks with time delay [31, 32]. They used stable lag synchronization and stable anticipatory synchronization between drive and response system to identify the unknown complex networks with time delay, respectively. In their studies, the adaptive control technique was used to make the network achieve synchronization. They considered an unknown complex network as a drive system. In order to identify the topology and system parameters, they designed a response network with an adaptive controller. Based on Lyapunov theory, the unknown topology and the uncertain system parameters can be identified when the lag/anticipatory synchronization is achieved.

According to the existed works, this paper investigates a method to identify an unknown complex networks through anticipatory projective synchronization. Under Lyapunov stability theory and Barbǎlat lemma, the asymptotic identification of the topology can be guaranteed. It is different from most of the works above because the anticipatory projective synchronization has never been studied before. Meanwhile, the example in this paper’s numerical simulation is making two complex networks achieve anticipatory projective synchronization. It is unlike other papers whose examples are single network’s synchronization.

The rest of this paper is organized as following. Several preliminaries and lemmas will be given in section II. Section III introduces the main theory of this paper, and a numerical simulation is used to show the effectiveness of the method in section IV. Finally, the conclusions will be remarked in section V.

## Preliminaries

Consider an uncertain dynamical complex network with ***N*** different nodes which are n-dimensional dynamical units as follows:

$${\dot{x}}_{i}(t)=f_{i}(x_{i}(t),x_{i}(t-\tau_{1}),\alpha_{i})+c\sum_{j=1}^{N}a_{ij}H_{ij}(x_{j}(t-\tau_{2})),\;i=1,2,\ldots ,N.$$ (1)

Here *x*_*i*_(*t*) ∈ *R* ^*n*×1^, *i* = 1,2,…,*N* is the dynamical state vector, *α*_*i*_ ∈ *R* ^*m*×1^ is an unknown system parameters vector of node *i*. The known dynamical function of node *i* is *f*_*i*_: *R* ^*n*^ → *R* ^*n*^. The coupling strength *c* > 0 is a constant, and *H*_*ij*_ is a known nonlinear function which represents the inner-coupling between node *i* and node *j*. The uncertain coupling configuration matrix *A* = (*a*_*ij*_)_*N*×*N*_ represents the topological structure of the complex network. If there is a direct link from node *i* to node *j*, then *a*_*ij*_ = *a*_*ji*_ ≠ 0, otherwise *a*_*ij*_ = 0. *τ*_1_, *τ*_2_ are the time-varying delay, and the coupling delay from node *j* to node *i*, respectively. If the unknown system parameter *α*_*i*_ is linearly dependent on the *i*th node’s dynamical nonlinear function *f*_*i*_, then Eq (1) can be rewritten as follows.

$${\dot{x}}_{i}(t)=F_{i}(x_{i}(t),x_{i}(t-\tau_{1}))+G_{i}(x_{i}(t),x_{i}(t-\tau_{1}))\alpha_{i}+c\sum_{j=1}^{N}a_{ij}H_{ij}(x_{j}(t-\tau_{2})).$$ (2)

Here *F*_*i*_ ∈ *R* ^*n*×1^ and *G*_*i*_ ∈ *R* ^*n*×*m*^ are known functions of the *i*th node dynamical.

Throughout this paper, in order to prove the main theory, the following assumptions and lemmas should be required.

*Assumption 1*. There exists a nonnegative constant *M* ≥ 0 and a constant vector ***σ*** ∈ *R* ^*n*×*n*^, for *i* = 1,2,…,*N*, such that

$$\begin{array}{l} {\left\|f_{i}(x_{i}(t),x_{i}(t-\tau ),\alpha_{i})-\sigma f_{i}(y_{i}(t),y_{i}(t-\tau ),\beta_{i})\right\|}^{2}\\ \leq M({\left\|x_{i}(t)-\sigma y_{i}(t)\right\|}^{2}+{\left\|x_{i}(t-\tau )-\sigma y_{i}(t-\tau )\right\|}^{2}) \end{array}.$$ (3)

*Assumption 2*. For any vectors **x**,**y** ∈ **R** ^*n*×1^, there exist a nonnegative constants *L* and a constant vector ***σ*** ∈ *R* ^*n*×*n*^, for *i* = 1,2,…,*N*, about *H*_*ij*_ in Eq (2), one gets

$$\|H_{ij}(x)-\sigma H_{ij}(y)\|\leq L\left\|x-\sigma y\right\|.$$

*Assumption 3*. There exists a constant *μ* which can make a differentiable time-varying delay *τ*(*t*) satisfied the following equation.

$$0\leq \dot{\tau }(t)\leq \mu <1.$$ (4)

Obviously, assumption 3 holds when *τ*(*t*) is a constant like *τ*(*t*) = *τ*_1_ or *τ*(*t*) = *τ*_2_ for any value of *t*.

*Remark 1*. It is obviously to see that assumption 1 and 2 hold as long as $\frac{\partial f_{i}}{\partial x}$, $\frac{\partial H_{ij}}{\partial x}$ are uniformly bounded [33]. A chaotic system which has the form of Eq (2) can meet the conditions of assumption 1 and 2 such as Lorenz system, Chen system, Chua’s circuit and so on.

*Lemma 1*. For any vector **x**,**y** ∈ **R** ^*n*×1^, the matrix inequality 2**x** ^*T*^ **y** ≤ **x** ^*T*^ **x** + **y** ^*T*^ **y** holds.

*Definition 1*. The drive and response systems can achieve anticipatory projective synchronization if Eq (5) is established.

$$\underset{t\to \infty }{\lim}\left\|y_{i}(t-\tau_{d})-\sigma x_{i}(t)\right\|=0.$$ (5)

Here *x*_*i*_(*t*) and *y*_*i*_(*t*) are the states vectors of drive and response system, respectively. The constant vector ***σ*** = *diag*(*σ*_1_,*σ*_2_,…,*σ*_*N*_) is the known scale factor of projective synchronization. *τ*_*d*_ is a positive anticipatory time.

## Main Theory

It denotes that *x*(*t*−*τ*) = *x* ^(*τ*)^ in the following to avoid any possible confusion. Then the drive system can be rewritten as:

$${\dot{x}}_{i}=F_{i}(x_{i},x_{i}^{\left(\tau_{1}\right)})+G_{i}(x_{i},x_{i}^{\left(\tau_{1}\right)})\alpha_{i}+c\sum_{j=1}^{N}a_{ij}H_{ij}(x_{j}^{\left(\tau_{2}\right)}).$$ (6)

In order to identify *α*_*i*_ and *a*_*ij*_ in complex network Eq (6), another complex network with input controller *u*_*i*_(*t*) is designed as response system. It can be described as follows:

$${\dot{y}}_{i}^{\left(\tau_{d}\right)}=F_{i}(y_{i}^{\left(\tau_{d}\right)},y_{i}^{\left(\tau_{d}+\tau_{1}\right)})+G_{i}(y_{i}^{\left(\tau_{d}\right)},y_{i}^{\left(\tau_{d}+\tau_{1}\right)})\beta_{i}^{\left(\tau_{d}\right)}+c\sum_{j=1}^{N}b_{ij}^{\left(\tau_{d}\right)}H_{ij}(y_{j}^{\left(\tau_{d}+\tau_{2}\right)})+u_{i}(t).$$ (7)

where $\beta_{i}^{\left(\tau_{d}\right)}$, $b_{ij}^{\left(\tau_{d}\right)}$ is changed by time *t* linearly and they can be estimated or measured. If $e_{i}=y_{i}^{\left(\tau_{d}\right)}-\sigma_{i}x_{i}$ is the error system between drive and response network, then Eq (6) and Eq (7) can achieve anticipatory synchronization when the following equation is established:

$$\underset{t\to \infty }{\lim}\left\|e_{i}\right\|=0.$$ (8)

*Theorem 1*. Under the assumption 1, 2 and 3, if the input controller *u*_*i*_(*t*) is chosen as Eq (9) and feedback gains are given as Eq (10), the uncertain system parameter vector *α*_*i*_ and unknown topology *a*_*ij*_ in Eq (6) can be identified by the estimated value $\beta_{i}^{\left(\tau_{d}\right)}$ and $b_{ij}^{\left(\tau_{d}\right)}$ in Eq (7)

$$u_{i}=-\sigma_{0}\tau_{d}e_{i}.$$ (9)

$$\left\{ \begin{array}{l} {\dot{\beta }}_{i}^{\left(\tau_{d}\right)}=-\omega G_{i}^{T}(y_{i}^{\left(\tau_{d}\right)},y_{i}^{\left(\tau_{1}+\tau_{d}\right)})e_{i}\\ {\dot{b}}_{ij}^{\left(\tau_{d}\right)}=-\xi e_{i}^{T}H_{ij}(y_{j}^{\left(\tau_{2}+\tau_{d}\right)})\\ {\dot{\tau }}_{d}=\psi \sigma_{0}e_{i}^{T}e_{i} \end{array}\right..$$ (10)

where *ω*, *ξ*, *ψ* are the positive constants, and *σ*_0_ = |***σ***| = |diag(*σ*_1_,*σ*_2_,…,*σ*_N_)|.

*Proof*. If uncertain system parameter *α*_*i*_ and unknown topology *a*_*ij*_ in Eq (6) can be identified by $\beta_{i}^{\left(\tau_{d}\right)}$ and $b_{ij}^{\left(\tau_{d}\right)}$ in Eq (7), then the following conditions should be guaranteed when $\underset{t\to \infty }{\lim}\left\|e_{i}\right\|=0$: $P_{i}=(\beta_{i}^{\left(\tau_{d}\right)}-\alpha_{i})=0$ and $Q_{ij}=(b_{ij}^{\left(\tau_{d}\right)}-a_{ij})=0$. Here *P*_*i*_ is the error system of parameters, *Q*_*ij*_ is the error system of topology.

Considering the input controller as Eq (9), the error system can be described as:

$$\begin{array}{l} {\dot{e}}_{i}=F_{i}(y_{i}^{\left(\tau_{d}\right)},y_{i}^{\left(\tau_{1}+\tau_{d}\right)})+G_{i}(y_{i}^{\left(\tau_{d}\right)},y_{i}^{\left(\tau_{1}+\tau_{d}\right)})\beta_{i}^{\left(\tau_{d}\right)}-\sigma_{i}F_{i}(x_{i},x_{i}^{\left(\tau_{1}\right)})-\sigma_{i}G_{i}(x_{i},x_{i}^{\left(\tau_{1}\right)})\alpha_{i}\\ \;+c\sum_{j=1}^{N}b_{ij}^{\left(\tau_{d}\right)}H_{ij}(y_{j}{}^{\left(\tau_{2}+\tau_{d}\right)})-c\sigma_{i}\sum_{j=1}^{N}a_{ij}H_{ij}(x_{j}{}^{\left(\tau_{2}\right)})+u_{i}\\ \;=F_{i}(y_{i}^{\left(\tau_{d}\right)},y_{i}^{\left(\tau_{1}+\tau_{d}\right)})+G_{i}(y_{i}^{\left(\tau_{d}\right)},y_{i}^{\left(\tau_{1}+\tau_{d}\right)})(P_{i}+\alpha_{i})-\sigma_{i}F_{i}(x_{i},x_{i}^{\left(\tau_{1}\right)})-\sigma_{i}G_{i}(x_{i},x_{i}^{\left(\tau_{1}\right)})\alpha_{i}\\ \;+c\sum_{j=1}^{N}\left(Q_{ij}+a_{ij})H_{ij}(y_{j}{}^{\left(\tau_{2}+\tau_{d}\right)}\right)-c\sigma_{i}\sum_{j=1}^{N}a_{ij}H_{ij}(x_{j}{}^{\left(\tau_{2}\right)})+u_{i}\\ \;=f_{i}(y_{i}^{\left(\tau_{d}\right)},y_{i}^{\left(\tau_{1}+\tau_{d}\right)},\alpha_{i})-\sigma_{i}f_{i}(x_{i},x_{i}^{\left(\tau_{1}\right)},\alpha_{i})+G_{i}(y_{i}^{\left(\tau_{d}\right)},y_{i}^{\left(\tau_{1}+\tau_{d}\right)})P_{i}\\ \;+c\sum_{j=1}^{N}Q_{ij}H_{ij}(y_{j}{}^{\left(\tau_{2}+\tau_{d}\right)})+c\sum_{j=1}^{N}a_{ij}H_{ij}(y_{j}{}^{\left(\tau_{2}+\tau_{d}\right)})-c\sum_{j=1}^{N}a_{ij}\sigma_{i}H_{ij}(x_{j}{}^{\left(\tau_{2}\right)})+u_{i} \end{array}.$$ (11)

Consider the following Lyapunov function:

$$\begin{array}{l} 2V(t)=\sum_{i=1}^{N}e_{i}^{T}e_{i}+\frac{1}{\omega }\sum_{i=1}^{N}P_{i}^{T}P_{i}+\frac{1}{\xi }\sum_{i=1}^{N}\sum_{j=1}^{N}Q_{ij}^{2}+\frac{1}{\psi }{\left(\tau_{d}-\tau_{0}\right)}^{2}\\ \;+\frac{M}{1-\mu }\sum_{i=1}^{N}\int_{t-\tau_{1}}^{t}e_{i}^{T}(z)e_{i}(z)dz+k\sum_{j=1}^{N}\int_{t-\tau_{2}}^{t}e_{j}^{T}(s)e_{j}(s)ds \end{array}.$$ (12)

Here *τ*_0_ > 0, *k* > 0 are positive constants need to be decided. Differentiating *V*(*t*) about time *t* along the solution of Eq (12), under control gains as Eq (10), one has

$$\begin{array}{l} \dot{V}=\sum_{i=1}^{N}e_{i}^{T}{\dot{e}}_{i}+\frac{1}{\omega }\sum_{i=1}^{N}P_{i}^{T}{\dot{\beta }}_{i}+\frac{1}{\xi }\sum_{i=1}^{N}\sum_{j=1}^{N}Q_{ij}{\dot{b}}_{ij}+\frac{1}{\psi }(\tau_{d}-\tau_{0}){\dot{\tau }}_{d}\\ \;+\frac{M}{2(1-\mu )}\sum_{i=1}^{N}\left[e_{i}^{T}e_{i}-(1-{\dot{\tau }}_{1})e_{i}^{(\tau_{1})T}e_{i}^{\left(\tau_{1}\right)}\right]+\frac{k}{2}\sum_{j=1}^{N}\left[e_{j}^{T}e_{j}-(1-{\dot{\tau }}_{2})e_{j}^{(\tau_{2})T}e_{j}^{\left(\tau_{2}\right)}\right]\\ \;=\sum_{i=1}^{N}e_{i}^{T}[f_{i}(y_{i}^{\left(\tau_{d}\right)},y_{i}^{\left(\tau_{1}+\tau_{d}\right)},\alpha_{i})-\sigma_{i}f_{i}(x_{i},x_{i}^{\left(\tau_{1}\right)},\alpha_{i})]+c\sum_{i=1}^{N}\sum_{j=1}^{N}e_{i}^{T}a_{ij}[H_{ij}(y_{j}{}^{\left(\tau_{2}+\tau_{d}\right)})-\sigma_{i}H_{ij}(x_{j}{}^{\left(\tau_{2}\right)})]\\ \;+\sum_{i=1}^{N}e_{i}^{T}[G_{i}(y_{i}^{\left(\tau_{d}\right)},y_{i}^{\left(\tau_{1}+\tau_{d}\right)})P_{i}]+c\sum_{i=1}^{N}\sum_{j=1}^{N}e_{i}^{T}Q_{ij}H_{ij}(y_{j}{}^{\left(\tau_{2}+\tau_{d}\right)})-\sum_{i=1}^{N}e_{i}^{T}u_{i}\\ \;+\frac{1}{\omega }\sum_{i=1}^{N}P_{i}^{T}[-\omega G_{i}^{T}(y_{i}^{\left(\tau_{d}\right)},y_{i}^{\left(\tau_{1}+\tau_{d}\right)})e_{i}]+\frac{1}{\xi }\sum_{i=1}^{N}\sum_{j=1}^{N}Q_{ij}[-c\xi e_{i}^{T}H_{ij}(y_{j}^{\left(\tau_{2}+\tau_{d}\right)})]+\frac{1}{\psi }(\tau_{d}-\tau_{0})(\psi \sigma_{0}e_{i}^{T}e_{i})\\ \;+\frac{M}{2(1-\mu )}\sum_{i=1}^{N}\left[e_{i}^{T}e_{i}-(1-{\dot{\tau }}_{1})e_{i}^{(\tau_{1})T}e_{i}^{\left(\tau_{1}\right)}\right]+\frac{k}{2}\sum_{j=1}^{N}\left[e_{j}^{T}e_{j}-(1-{\dot{\tau }}_{2})e_{j}^{(\tau_{2})T}e_{j}^{\left(\tau_{2}\right)}\right]\\ \;=\sum_{i=1}^{N}e_{i}^{T}[f_{i}(y_{i}^{\left(\tau_{d}\right)},y_{i}^{\left(\tau_{1}+\tau_{d}\right)},\alpha_{i})-\sigma_{i}f_{i}(x_{i},x_{i}^{\left(\tau_{1}\right)},\alpha_{i})]+c\sum_{i=1}^{N}\sum_{j=1}^{N}e_{i}^{T}a_{ij}[H_{ij}(y_{j}{}^{\left(\tau_{2}+\tau_{d}\right)})-\sigma_{i}H_{ij}(x_{j}{}^{\left(\tau_{2}\right)})]\\ \;-\tau_{0}\sigma_{i}e_{i}^{T}e_{i}+\frac{M}{2(1-\mu )}\sum_{i=1}^{N}e_{i}^{T}e_{i}-\frac{M(1-{\dot{\tau }}_{1})}{2(1-\mu )}\sum_{i=1}^{N}e_{i}^{(\tau_{1})T}e_{i}^{\left(\tau_{1}\right)}+\frac{k}{2}\sum_{j=1}^{N}\left[e_{j}^{T}e_{j}-(1-{\dot{\tau }}_{2})e_{j}^{(\tau_{2})T}e_{j}^{\left(\tau_{2}\right)}\right]\;. \end{array}$$

Under the assumptions 1, 2 and lemma 1, one has

$$e_{i}^{T}[f_{i}(y_{i}^{\left(\tau_{d}\right)},y_{i}^{\left(\tau_{1}+\tau_{d}\right)},\alpha_{i})-\sigma_{i}f_{i}(x_{i},x_{i}^{\left(\tau_{1}\right)},\alpha_{i})]\leq \frac{M+1}{2}e_{i}^{T}e_{i}+\frac{M}{2}e_{i}^{(\tau_{1})T}e_{i}^{\left(\tau_{1}\right)},$$

$$a_{ij}e_{i}^{T}[H_{ij}(y_{j}{}^{\left(\tau_{2}+\tau_{d}\right)})-\sigma_{i}H_{ij}(x_{j}{}^{\left(\tau_{2}\right)})]\leq \frac{L\left|a_{ij}\right|}{2}(e_{i}^{T}e_{i}+e_{j}^{(\tau_{2})T}e_{j}^{\left(\tau_{2}\right)}).$$

It denotes that $E^{T}(t)=[e_{1}^{T}(t),e_{2}^{T}(t),\ldots ,e_{N}^{T}(t)]$, *a*_*M*_ = max_*i*,*j* = 1,2,…,*N*_ {|*a*_*ij*_|}, then one has

$$\begin{array}{l} \dot{V}\leq (\frac{M+1}{2}+\frac{M+1}{2(1-\mu )}+\frac{La_{M}}{2}-\tau_{0}\sigma_{0})\sum_{i=1}^{N}e_{i}^{T}e_{i}+\sum_{i=1}^{N}\frac{-M(\mu -{\dot{\tau }}_{1})}{2(1-\mu )}e_{i}^{(\tau_{1})T}e_{i}^{\left(\tau_{1}\right)}\\ \;+\frac{k}{2}\sum_{j=1}^{N}\left[e_{j}^{T}e_{j}-(1-{\dot{\tau }}_{2})e_{j}^{(\tau_{2})T}e_{j}^{\left(\tau_{2}\right)}\right]+\frac{La_{M}}{2}\sum_{j=1}^{N}e_{j}^{(\tau_{2})T}e_{j}^{\left(\tau_{2}\right)}\\ \;\leq (\frac{M+1}{2}+\frac{M+1}{2(1-\mu )}+\frac{La_{M}}{2}+\frac{k}{2}-\tau_{0}\sigma_{0})E^{T}E\\ \;+\frac{M({\dot{\tau }}_{1}-\mu )}{2(1-\mu )}E^{(\tau_{1})T}E^{\left(\tau_{1}\right)}+\frac{k({\dot{\tau }}_{2}-1)+La_{M}}{2}E^{(\tau_{2})T}E^{\left(\tau_{2}\right)} \end{array}.$$

Obviously, there exist constants to let $\tau_{0}\geq \frac{M+1}{2\sigma_{0}}+\frac{M+1}{2\sigma_{0}(1-\mu )}+\frac{La_{M}}{2\sigma_{0}}+\frac{k}{2\sigma_{0}}$ and $k\geq \frac{La_{M}}{1-{\dot{\tau }}_{2}}$, according to assumption 3, the above inequality can be obtained as

$$\dot{V}\leq -E^{T}E\leq 0.$$ (13)

According Lyapunov theory, the system can achieve anticipatory projective synchronization as *t* → ∞. Consider about Eq (13), one has

$$0\leq \underset{t\to \infty }{\lim}\int_{0}^{t}E^{T}(s)E(s)ds\leq V(0)-\underset{t\to \infty }{\lim}V(t).$$ (14)

Consider about Eq (12) and Eq (13), the right part of Eq (14) is bounded because both *V*(0) and *V*(*t*) are bounded. Moreover, *E*(*t*) is bounded. $\dot{e}(t)$ is existed and bounded because of Eq (11), thus according to the Barbǎlat’s lemma, one has $\underset{t\to \infty }{\lim}E(t)=0$, that is to say, $\underset{t\to \infty }{\lim}{\dot{e}}_{i}(t)=0$. When *t* → ∞, consider of Eq (11), according to [33], $y_{i}^{\left(\tau_{d}\right)}$ converges as

$$\varepsilon_{i}=\left\{y_{i}^{\left(\tau_{d}\right)}:G_{i}(y_{i}^{\left(\tau_{d}\right)},y_{i}^{\left(\tau_{d}+\tau_{1}\right)})P_{i}+c\sum_{j=1}^{N}Q_{ij}H_{ij}(y_{j}^{\left(\tau_{d}+\tau_{2}\right)})=0\right\},$$

because $G_{i}(y_{i}^{\left(\tau_{d}\right)},y_{i}^{\left(\tau_{d}+\tau_{1}\right)})\neq 0$, $H_{ij}(y_{j}^{\left(\tau_{d}+\tau_{2}\right)})\neq 0$, in order to make the following equation is established, one has *P*_*i*_ → 0, *Q*_*ij*_ → 0 as *t* → ∞.

$$G_{i}(y_{i}^{\left(\tau_{d}\right)},y_{i}^{\left(\tau_{d}+\tau_{1}\right)})P_{i}+c\sum_{j=1}^{N}Q_{ij}H_{ij}(y_{j}^{\left(\tau_{d}+\tau_{2}\right)})=0$$

Therefore, when *t* → ∞, the unknown system parameters and uncertain topology can be identified by the estimated value. The proof is complete.

## Examples

In this section, a numerical simulation is given to show the effectiveness of theorem 1 in section 3. This simulation chooses chaotic Lorenz system as the dynamics of each node. Lorenz system can be described as

$$f(x)=\left\{ \begin{array}{l} {\dot{x}}_{i1}=f(x_{i1})=v(x_{i2}-x_{i1})\\ {\dot{x}}_{i2}=f(x_{i2})=\alpha x_{i1}-x_{i1}x_{i3}-x_{i2}\\ {\dot{x}}_{i3}=f(x_{i3})=x_{i1}x_{i2}-ux_{i3} \end{array}\right..$$ (15)

When the parameters are chosen as *v* = 10, *α* = 28, *u* = 2.67, the Lorenz system Eq (15) is chaotic.

Consider a complex network consisting 6 identical nodes which dynamic function like Eq (15). The state vector of each node is ***x***_*i*_ = (*x*_1*i*_, *x*_2*i*_, *x*_3*i*_)^*T*^,*i* = 1,2,…,6. Because parameter *α* is linearly dependent on Lorenz function, then the drive complex network can be described as

$${\dot{x}}_{i}=F_{i}(x_{i},x_{i}^{\left(\tau_{1}\right)})+G_{i}(x_{i},x_{i}^{\left(\tau_{1}\right)})\alpha_{i}+c\sum_{j=1}^{6}a_{ij}H_{ij}(x_{j}^{\left(\tau_{2}\right)}).$$ (16)

Here $F_{i}(x)={\left(10(x_{i2}{}^{\left(\tau_{1}\right)}-x_{i1}{}^{\left(\tau_{1}\right)}),-x_{i1}{}^{\left(\tau_{1}\right)}x_{i3}{}^{\left(\tau_{1}\right)}-x_{i2}{}^{\left(\tau_{1}\right)},x_{i1}{}^{\left(\tau_{1}\right)}x_{i2}{}^{\left(\tau_{1}\right)}-2.67x_{i3}{}^{\left(\tau_{1}\right)}\right)}^{T}$, $G_{i}(x)={\left(0,x_{i1}{}^{\left(\tau_{1}\right)},0\right)}^{T}$, the network is constructed as a BA scale-free network. The coupling configuration matrix *A* = {*a*_*ij*_} and parameter *α*_*i*_ is unknown. In order to identify *α*_*i*_ and *A* in Eq (16), this paper designs a response complex network as:

$${\dot{y}}_{i}^{\left(\tau_{d}\right)}=F_{i}(y_{i},y_{i}^{\left(\tau_{1}\right)})+G_{i}(y_{i},y_{i}^{\left(\tau_{1}\right)})\beta_{i}^{\left(\tau_{d}\right)}+c\sum_{j=1}^{6}b_{ij}{}^{\left(\tau_{d}\right)}H_{ij}(y_{j}^{\left(\tau_{2}\right)}).$$ (17)

which dynamical function *F* and *G* are the same as drive system Eq (14), the value of $\beta_{i}^{\left(\tau_{d}\right)}$ and $b_{ij}^{\left(\tau_{d}\right)}$ is given randomly, the other parameters are the same as the drive network Eq (17).

In simulation, the estimated value of uncertain parameters is given as *α*_*i*_ = (21,23,24,25,26,28), the estimated value of known coupling configuration matrix is given as

$$A=\left[\begin{array}{cccccc} 0 & 1 & -3 & 5 & -2 & 7\\ 1 & 0 & -10 & -1 & 0 & -4\\ -3 & -10 & 0 & 3 & 10 & 4\\ 5 & -1 & 3 & 0 & -1 & -1\\ -2 & 0 & 10 & -1 & 0 & 0\\ 7 & -4 & 4 & -1 & 0 & 0 \end{array}\right].$$

The scale factor of projective synchronization is *σ*_*i*_ = *diag*(0.1,0.2,0.3,0.4,0.5,0.6), the other parameters are the same as the drive network Eq (17), the error system between drive and response network is described as *E*_*i*_(*t*) = (*e*_*i*1_(*t*),*e*_*i*2_(*t*),*e*_*i*3_(*t*))^*T*^, the adaptive feedback are given as *ω* = *ξ* = *ψ* = 1, the initial values of the drive and response network are given randomly. In order to show the effectiveness of proposed method, after *t* = 500, for *i* = 1,2,…,6, the estimated value are changed to $\alpha_{i}^{\prime }=(20.5,23.1,24.2,25.3,25.1,28.8)$,

$$A^{\prime }=\left[\begin{array}{cccccc} 0 & -2 & 16 & 0 & -10 & -4\\ -2 & 0 & 12 & 10 & 0 & 3\\ 16 & 12 & 0 & -16 & -12 & 2\\ 0 & 10 & -16 & 0 & -1 & -1\\ -10 & 0 & -12 & -1 & 0 & 1\\ -4 & 3 & 2 & -1 & 1 & 0 \end{array}\right].$$

### Example 1. The identification of complex network without time delay

**Fig 1:**
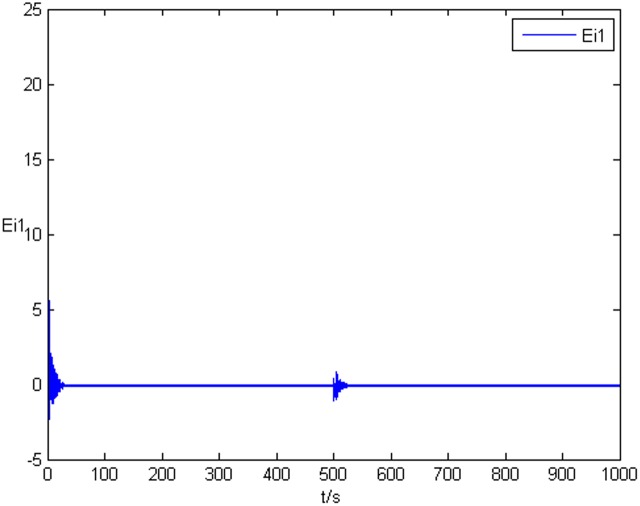
State value of the first components error system without time delay.

**Fig 2:**
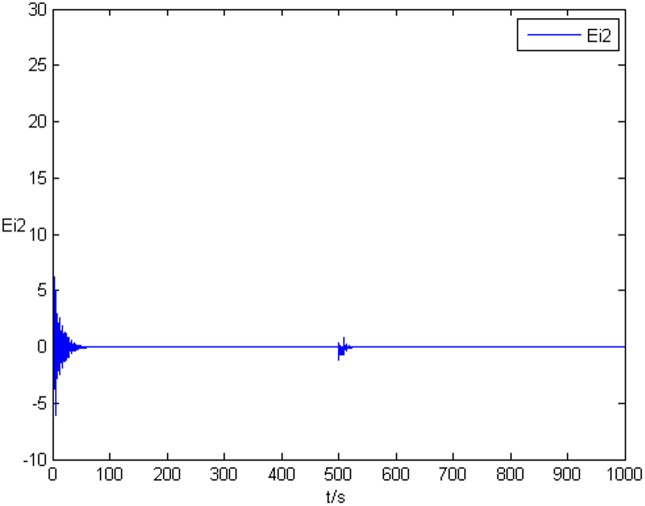
State value of the second components error system without time delay.

**Fig 3:**
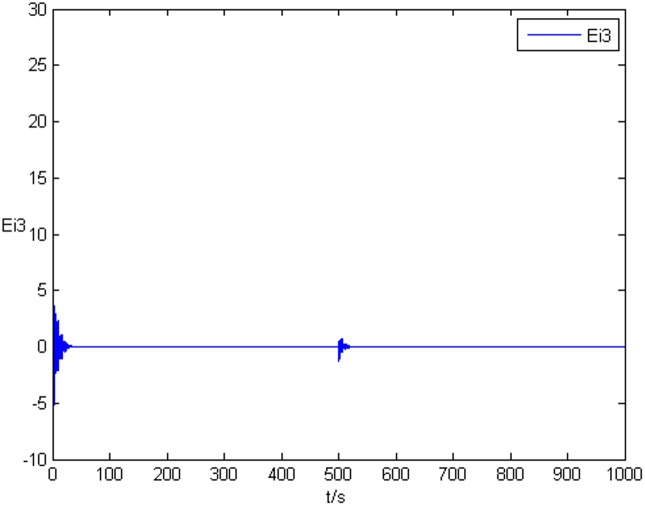
State value of the third components error system without time delay.

**Fig 4:**
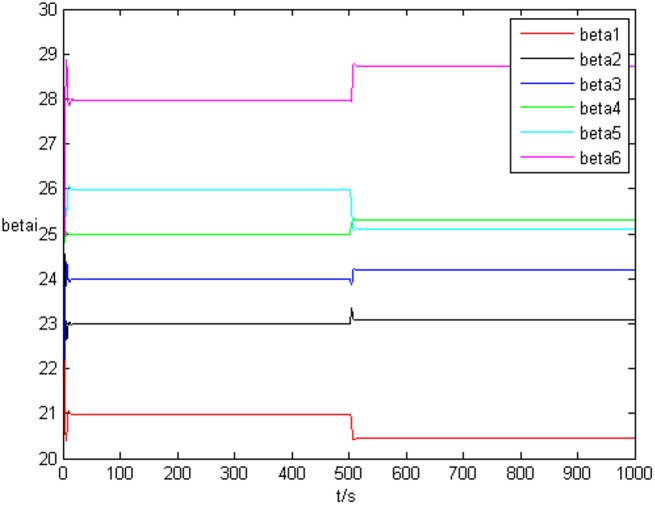
State value of system parameter without time delay.

**Fig 5:**
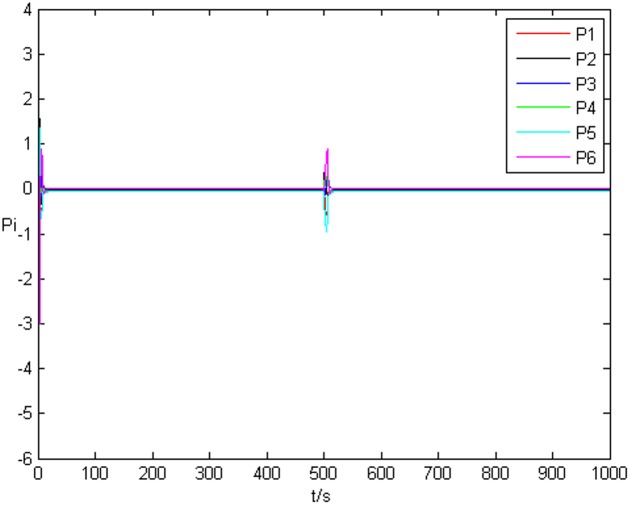
Error system of system parameter without time delay.

**Fig 6:**
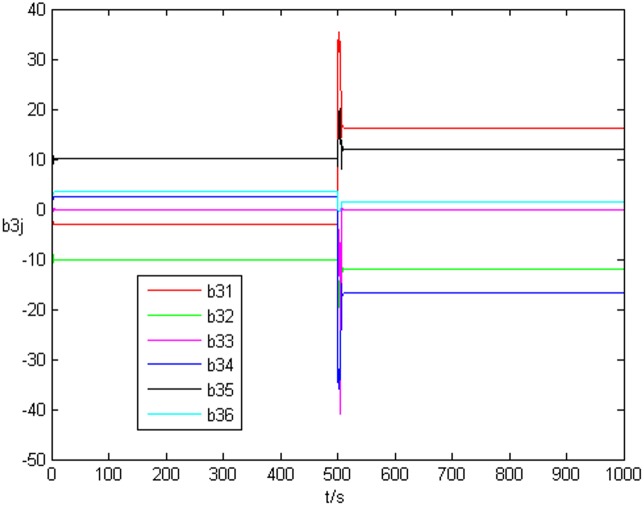
State value of the third node’s topology without time delay.

**Fig 7:**
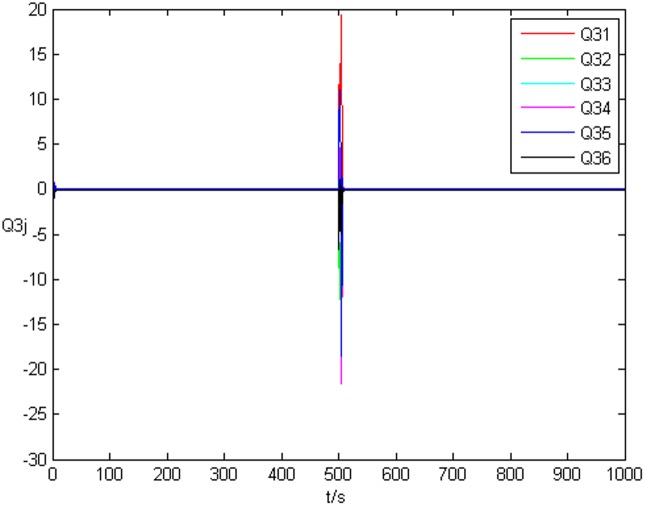
Error system of the third node’s topology without time delay.

If the drive and response complex networks have no time delay, that is to say, *τ*_1_ = *τ*_2_ = 0 in Eq (16) and Eq (17), the results of simulation are shown as follows. The figure of error system *E*_*i*_(*t*) = (*e*_*i*1_(*t*),*e*_*i*2_(*t*),*e*_*i*3_(*t*))^*T*^ is shown in Fig 1, Fig 2, and Fig 3, respectively. It can be seen that under the controller Eq (9) and the feedback controller Eq (10), the drive network and the response network can achieve anticipatory projective synchronization. When *t* > 500, under the proposed controller, the drive network and the response network can achieve anticipatory projective synchronization too. The process of identification about unknown system parameter is shown in Fig 4. According to the simulation results, *β*_*i*_(*t*) can achieve *α*_*i*_ when the system achieve anticipatory projective synchronization. After *t* > 500, even the estimated value is changed, *β*_*i*_(*t*) can also achieve $\alpha_{i}^{\prime }$ finally. Fig 5 shows the process of parameter’s error system *P*_*i*_. Fig 6 shows the identification process of uncertain topology of the third node *a*_3*j*_. It is easy to see that *a*_3*j*_ can be identified by the estimated value *b*_3*j*_ obviously when the anticipatory projective synchronization is achieved. After *t* > 500, *b*_3*j*_ can achieve $a_{3j}^{\prime }$ at last. Fig 7 shows the error system of topology *Q*_3*j*_. Thus, when the drive network and response network achieve anticipatory projective synchronization, the unknown system parameters and uncertain topology can be identified by the response network even they are changed during the identification process.

### Example 2. The identification of complex network with time delay

**Fig 8:**
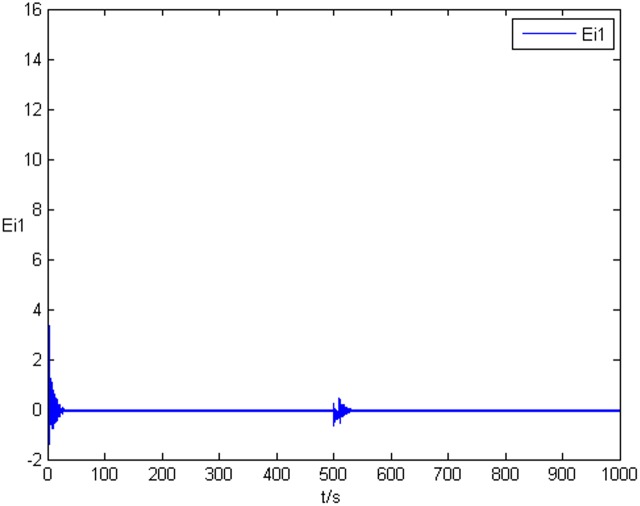
State value of the first components error system with time delay.

**Fig 9:**
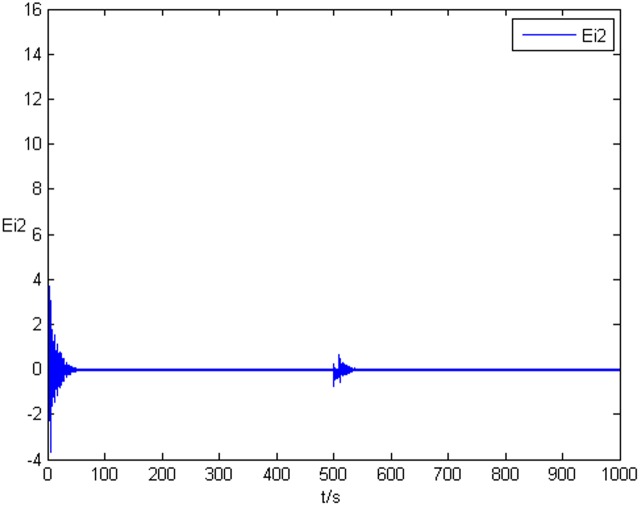
State value of the second components error system with time delay.

**Fig 10:**
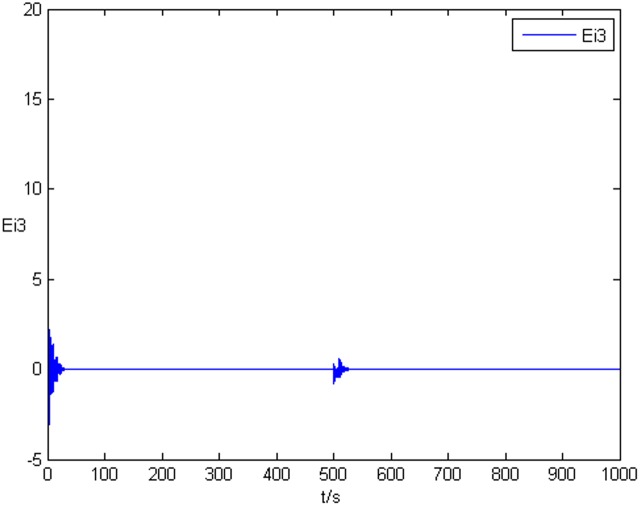
State value of the third components error system with time delay.

**Fig 11:**
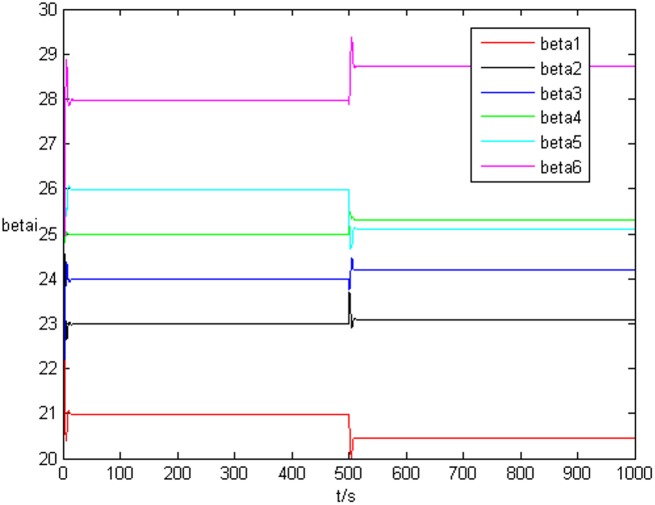
State value of system parameter with time delay.

**Fig 12:**
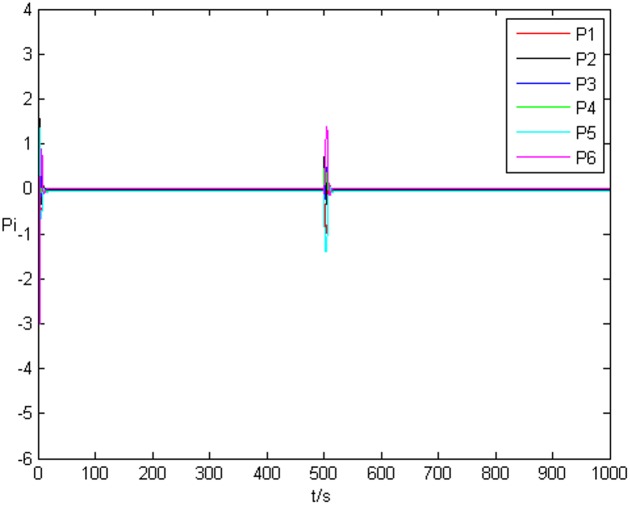
Error system of system parameter with time delay.

**Fig 13:**
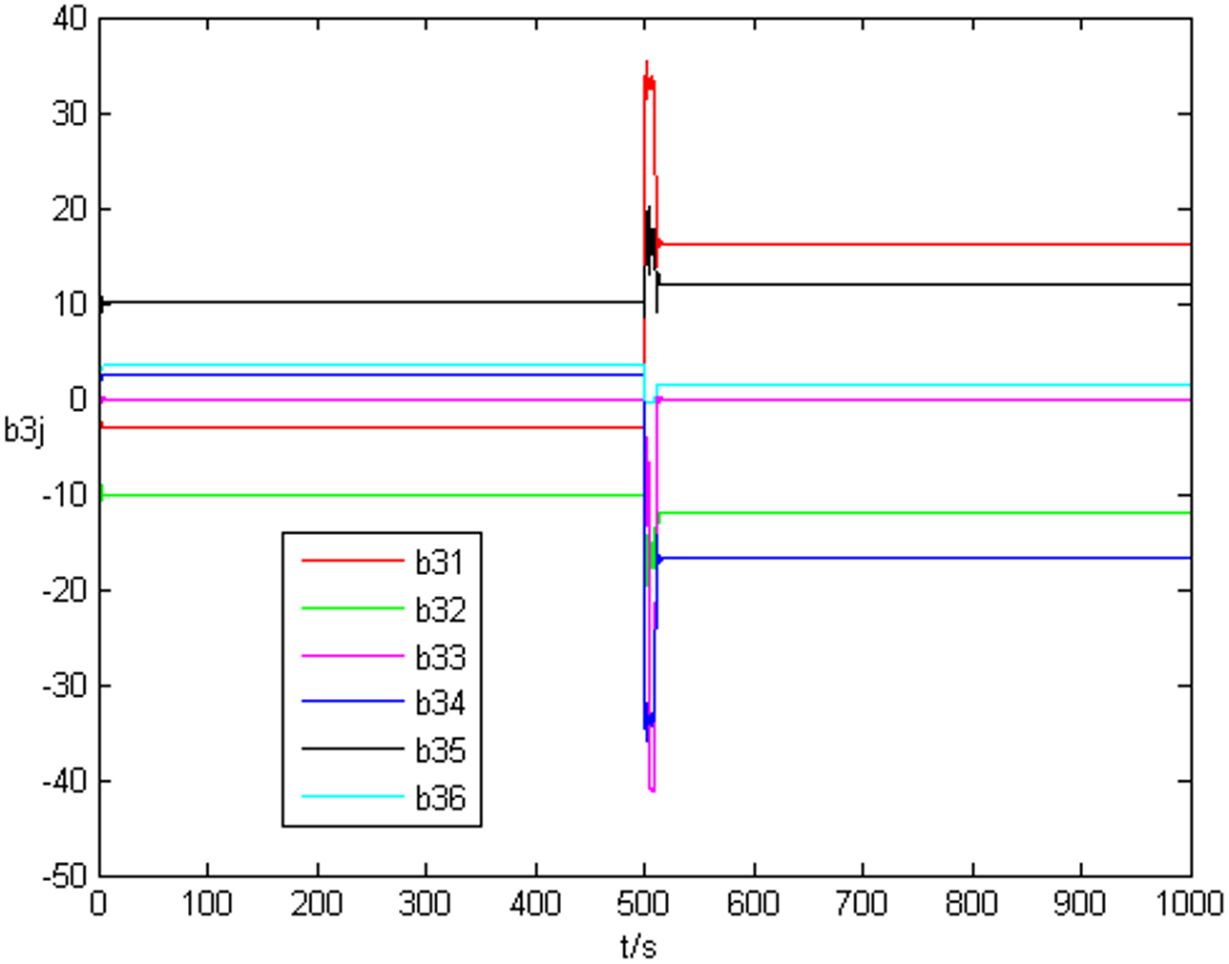
State value of the third node’s topology with time delay.

**Fig 14:**
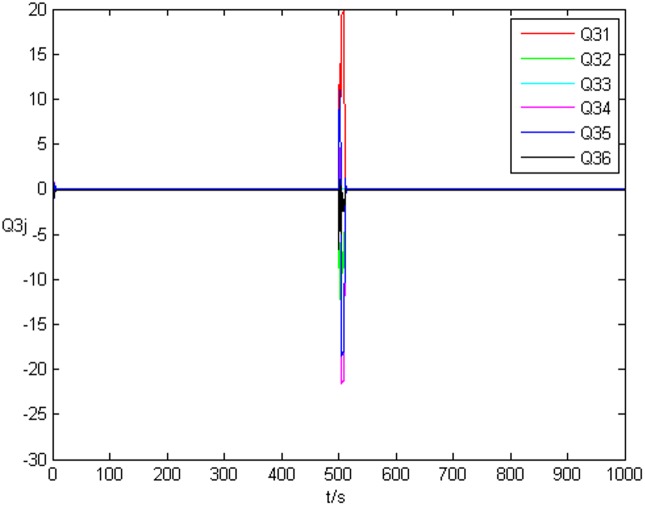
Error system of the third node’s topology with time delay.

If the drive and response complex networks have time delay as *τ*_1_ = 1.5, *τ*_2_ = 0.5 in Eq (16) and Eq (17), the simulation results are shown as follows. Figs 8–10 show the state of error system. Obviously, when complex networks have time delays, the system can achieve anticipatory projective synchronization under the controller Eq (9) and Eq (10). because *E*_*i*1_ = *E*_*i*2_ = *E*_*i*3_ = 0 when *t* → ∞. Fig 11 shows the changing of known system parameters when the drive and response networks have time delays. The results show that when the system achieves anticipatory projective synchronization, the known parameters *β*_*i*_ in response network can achieve unknown parameters *α*_*i*_ even *α*_*i*_ is changed after *t* > 500. Fig 12 shows the state of error system about unknown parameters *P*_*i*_. The simulation results of uncertain topological identification of *b*_3*j*_ and the state of error system about uncertain topology *Q*_3*j*_ are shown in Fig 13 and Fig 14, respectively.

## Conclusions

This paper investigated a method to identify complex network with unknown system parameters and uncertain topological structure. A response complex network, which parameters and topology can be estimated or can be measured, is designed to achieve anticipatory projective synchronization with the unknown drive complex network. When the synchronization is achieved, the parameters and topology of response network can be changed to equal with the parameters and topology in drive network. That is to say, the unknown parameters and uncertain topology of drive complex network can be identified by the response complex network. Comparing to the other existing work, this paper used anticipatory projective synchronization which hasn’t been appeared in other papers. The input controller’s adaptive feedback is reflected by the anticipatory projective scale factor’s vector. It is different from other papers whose adaptive feedback’s parameters are always constants. The simulation in this paper uses outer synchronization between two independent complex networks, and other papers often use inner synchronization in one complex network.
